# 4-(1*H*-2,3-Dihydro­naphtho­[1,8-*de*][1,3,2]di­aza­borinin-2-yl)-1-ethylpyridin-1-ium iodide

**DOI:** 10.1107/S2414314624003626

**Published:** 2024-04-26

**Authors:** Shu Hashimoto, Tsunehisa Okuno

**Affiliations:** a Department of Systems Engineering, Wakayama University, Sakaedani, Wakayama, 640-8510, Japan; University of Antofagasta, Chile

**Keywords:** crystal structure, pyridinium ion, Bdan

## Abstract

The title compound, C_17_H_17_BN_3_I, is a type of di­aza­borinane featuring substitution at the 1, 2, and 3 positions of the nitro­gen–boron six-membered heterocycle. The organic mol­ecule has a planar structure, the dihedral angle between the pyridyl ring and the fused ring system being 3.46 (4)°. In the crystal, mol­ecules are stacked in a head-to-tail manner. The iodide ion makes close contacts with three organic mol­ecules and supports the alternating stack.

## Abstract 2

The title compound is a di­aza­borinane featuring substitution at the 1, 2, and 3 positions of the nitro­gen–boron six-membered heterocycle. In the crystal, mol­ecules are stacked in a head-to-tail manner. The iodide ion makes close contacts with three organic mol­ecules and supports the alternating stack.

## Graphical abstract

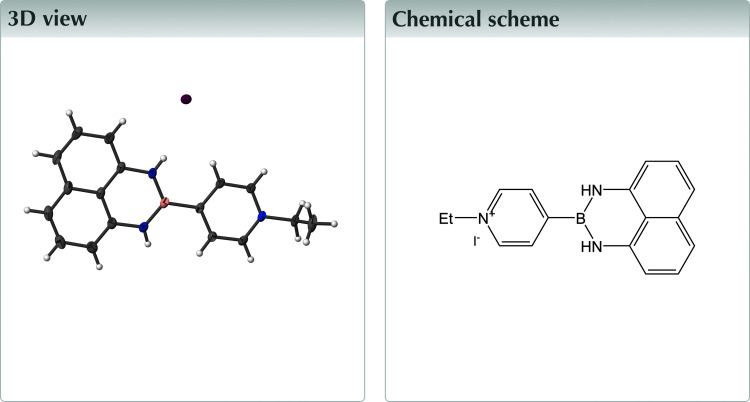

## Structure description

**Figure 1:**
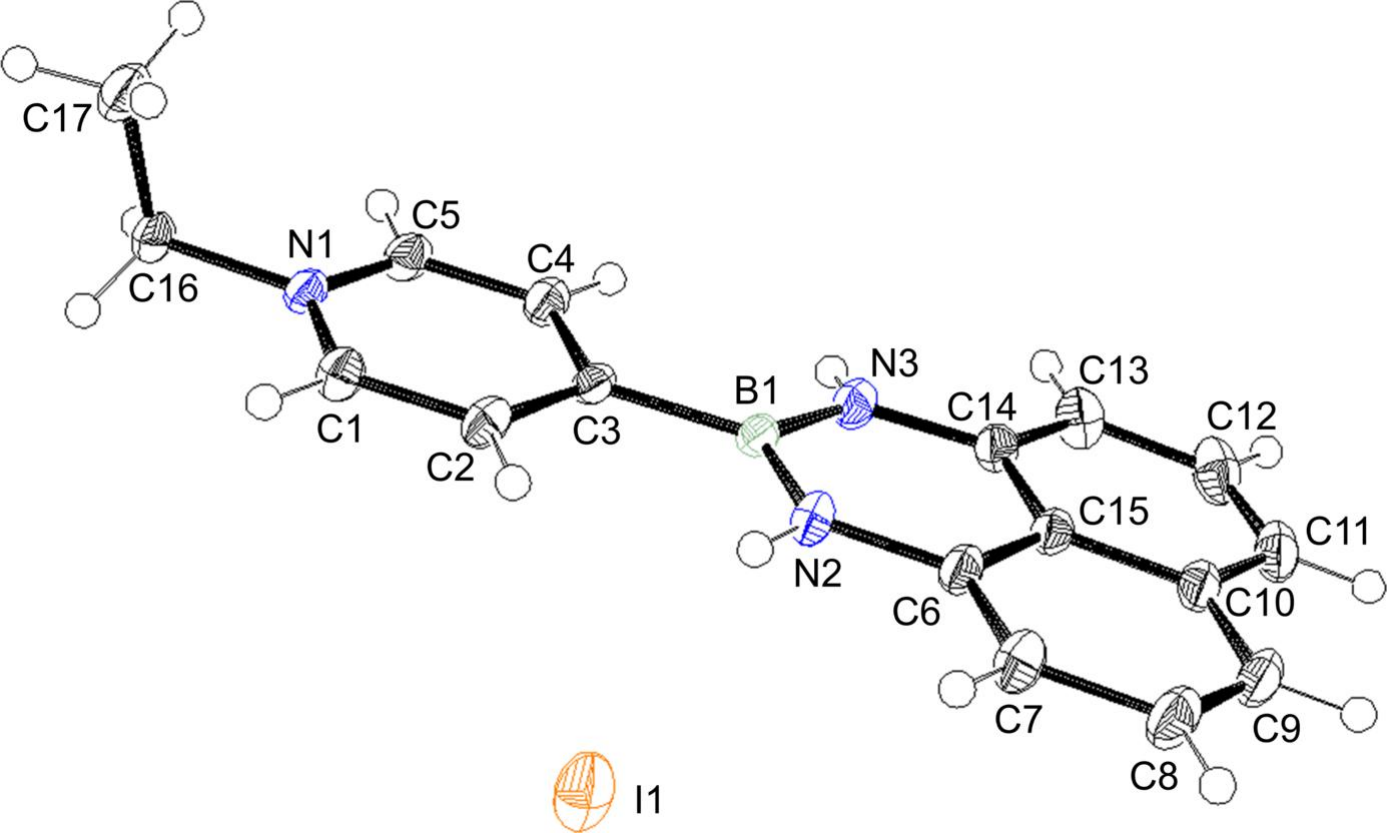
The title compound with the atom-numbering scheme. Displacement ellipsoids are drawn at the 50% probability level and H atoms are shown as small spheres of arbitrary radii.

The title compound (Fig. 1 ▸) is a type of di­aza­borinane featuring substitution at 1, 2, and 3 positions in the nitro­gen-boron six-membered heterocycle. Di­aza­borinanes have been found to stabilize organic radicals (LaPorte *et al.*, 2023 ▸). The hydrated polymorph of the title compound was reported by Hashimoto *et al.* (2024 ▸).

**Figure 2:**
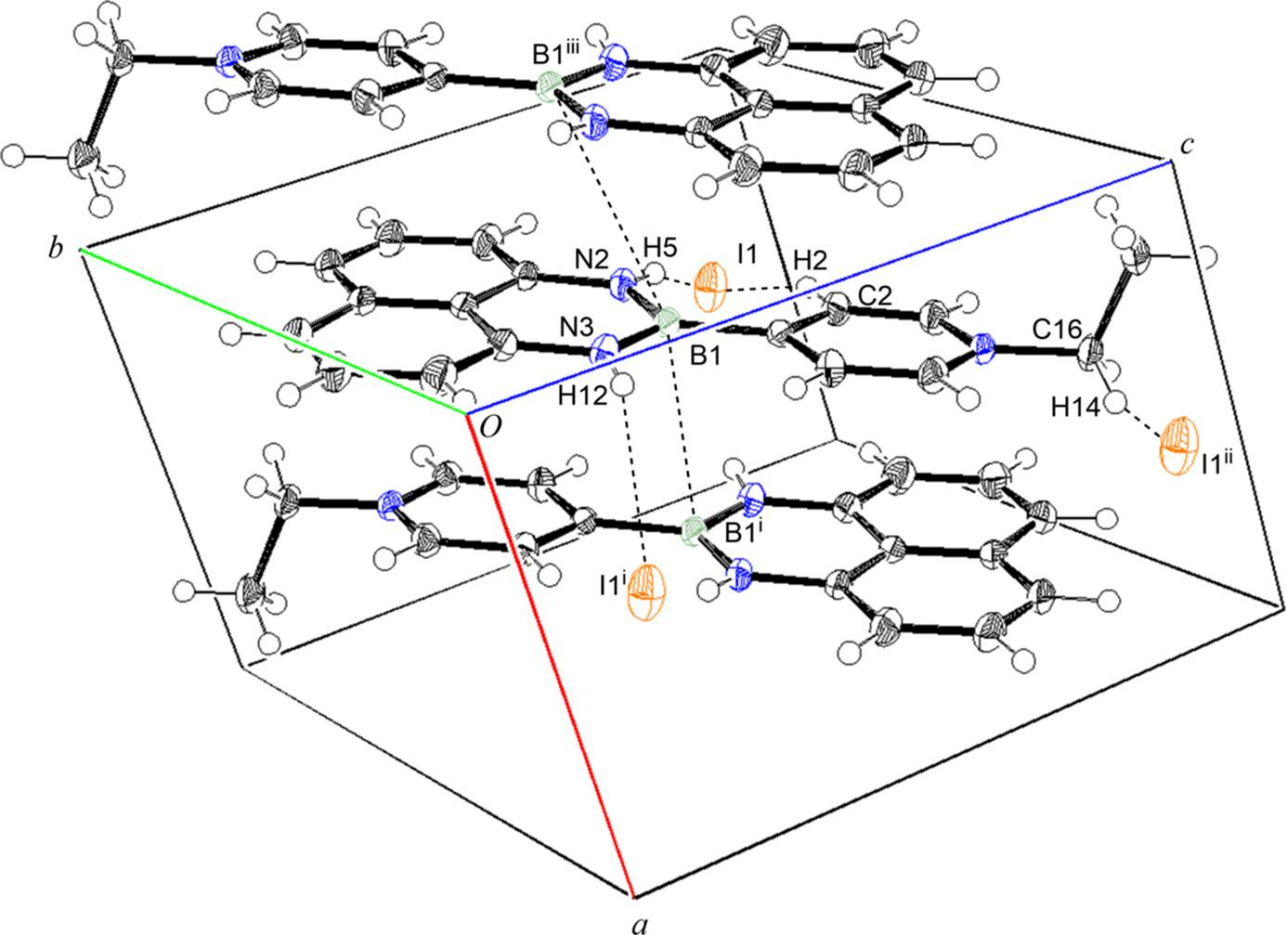
Inter­molecular inter­actions in the title compound [symmetry codes: (i) −*x* + 1, −*y* + 1, −*z* + 1; (ii) *x*, *y* − 1, *z*; (iii) −*x*, −*y* + 1, −*z* + 1].

The organic unit has a planar structure, with a dihedral angle between the N1/C1—C5 pyridyl ring and the N2/N3/C6–C15/B1 ring system of 3.46 (4)°. The organic unit has the similar structure to those previously reported (Akerman *et al.*, 2011 ▸; Slabber *et al.*, 2011 ▸). The ethyl group on the nitro­gen atom has an out-of-plane conformation. In the crystal, the organic unit forms alternating stacks in a head-to-tail manner along the *a* axis, as shown in Fig. 2 ▸, where the B1⋯·B1^i^ and B1⋯·B1^iii^ distances are 3.380 (3) and 3.793 (3) Å, respectively [symmetry codes:(i) −*x* + 1, −*y* + 1, −*z* + 1; (iii) −*x*, −*y* + 1, −*z* + 1]. Three kinds of hydrogen bonds occur between the organic unit and the iodide ions, as summarized in Table 1 ▸, the with iodide ion being surrounded by three organic units. It supports the alternating stacking and connects neighboring stacks.

## Synthesis and crystallization

The precursor of the title compound, 2-(pyridin-4-yl)-2,3-di­hydro-1*H*-naphtho­[1,8-*de*][1,3,2] di­aza­borinine, **4PyBdan**, was prepared by condensation of 4-(4,4,5,5-tetra­methyl-1,3,2-dioxaborolan-2-yl)pyridine and 1,8-di­aminona­phthalene. A solution of 4-(4,4,5,5-tetra­methyl-1,3,2-dioxaborolan-2-yl)pyridine (0.20 g, 0.98 mmol) and 1,8-di­aminona­phthalene (0.20 g, 1.3 mmol) in dry toluene (50 ml) was refluxed for 24 h under an argon atmosphere. The solution was concentrated under reduced pressure. The residual solid was purified by column chromatography (SiO_2_, ethyl acetate) to give a yellow solid of **4PyBdan** (0.23 g) in 96% yield. ^1^H NMR (400 MHz, CDCl_3_): δ 6.44 (*br s*, 2H), 6.44 (*d*, *J* = 8.2 Hz, 2H), 7.09 (*d*, *J* = 8.2 Hz, 2H), 7.15 (*t*, *J* = 8.2 Hz, 2H), 7.50 (*d*, *J* = 6.0 Hz, 2H), 8.69 (*d*, *J* = 6.0 Hz, 2H).

A mixture of **4PyBdan** (0.15 g, 0.61 mmol) and iodo­ethane (3.0 ml, 37.7 mmol) in aceto­nitrile (24 ml) was stirred for 14 h under an argon atmosphere. The precipitate was filtered off and dried under vacuum to give the title compound (0.14 g) in 57% yield as a red solid. Single crystals of sufficient quality were obtained by recrystallization from aceto­nitrile.

## Refinement

Crystal data, data collection and structure refinement details are summarized in Table 2 ▸.

## Supplementary Material

Crystal structure: contains datablock(s) I. DOI: 10.1107/S2414314624003626/bx4026sup1.cif

Structure factors: contains datablock(s) I. DOI: 10.1107/S2414314624003626/bx4026Isup2.hkl

Supporting information file. DOI: 10.1107/S2414314624003626/bx4026Isup3.cml

CCDC reference: 2349942

Additional supporting information:  crystallographic information; 3D view; checkCIF report

## Figures and Tables

**Table 1:** Hydrogen-bond geometry (Å, °)

*D*—H⋯*A*	*D*—H	H⋯*A*	*D*⋯*A*	*D*—H⋯*A*
N2—H5⋯I1	0.81 (2)	2.96 (2)	3.7260 (13)	157.2 (18)
C2—H2⋯I1	0.93	3.16 (1)	4.0483 (14)	161 (1)
N3—H12⋯I1^i^	0.82 (2)	3.02 (2)	3.7453 (12)	148.7 (18)
C16—H14⋯I1^ii^	0.97	3.07 (1)	3.8981 (15)	144 (1)

Symmetry codes: (i) 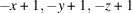 ; (ii) 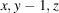 .

**Table 2:** Experimental details

Crystal data	
Chemical formula	C_17_H_17_BN_3_^+^·I^−^
*M*_r_	401.04
Crystal system, space group	Triclinic, *P* 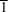
Temperature (K)	293
*a*, *b*, *c* (Å)	7.0800 (3), 10.6304 (3), 11.0650 (3)
α, β, γ (°)	89.715 (2), 79.711 (3), 89.598 (2)
*V* (Å^3^)	819.37 (5)
*Z*	2
Radiation type	Mo *K*α
μ (mm^−1^)	1.95
Crystal size (mm)	0.1 × 0.05 × 0.03

Data collection	
Diffractometer	XtaLAB AFC10 (RCD3): fixed-χ single
Absorption correction	Multi-scan (*CrysAlis PRO*; Rigaku OD, 2020 ▸)
*T*_min_, *T*_max_	0.942, 1.000
No. of measured, independent and observed [*I* > 2σ(*I*)] reflections	12190, 4404, 4088
*R*_int_	0.016
(sin θ/λ)_max_ (Å^−1^)	0.737

Refinement	
*R*[*F* ^2^ > 2σ(*F* ^2^)], *wR*(*F* ^2^), *S*	0.020, 0.052, 1.07
No. of reflections	4404
No. of parameters	208
H-atom treatment	H atoms treated by a mixture of independent and constrained refinement
Δρ_max_, Δρ_min_ (e Å^−3^)	0.78, −0.29

Computer programs: *CrysAlis PRO* (Rigaku OD, 2020 ▸), *SHELXT2014/4* (Sheldrick, 2015*a* ▸), *SHELXL2014/7* (Sheldrick, 2015*b* ▸) and *OLEX2* (Dolomanov *et al.*, 2009 ▸).

## full crystallographic data

### Crystal data

**Table:**

C_17_H_17_BN_3_^+^·I^−^	*Z* = 2
*M**_r_* = 401.04	*F*(000) = 396
Triclinic, *P*1	*D*_x_ = 1.626 Mg m^−^^3^
*a* = 7.0800 (3) Å	Mo *K*α radiation, λ = 0.71073 Å
*b* = 10.6304 (3) Å	Cell parameters from 7826 reflections
*c* = 11.0650 (3) Å	θ = 3.8–31.4°
α = 89.715 (2)°	µ = 1.95 mm^−^^1^
β = 79.711 (3)°	*T* = 293 K
γ = 89.598 (2)°	Plate, clear red
*V* = 819.37 (5) Å^3^	0.1 × 0.05 × 0.03 mm

### Data collection

**Table:**

XtaLAB AFC10 (RCD3): fixed-χ single diffractometer	4404 independent reflections
Radiation source: Rotating-anode X-ray tube, Rigaku (Mo) X-ray Source	4088 reflections with *I* > 2σ(*I*)
Mirror monochromator	*R*_int_ = 0.016
Detector resolution: 10.0000 pixels mm^-1^	θ_max_ = 31.6°, θ_min_ = 3.7°
ω scans	*h* = −7→10
Absorption correction: multi-scan (CrysAlisPro; Rigaku OD, 2020)	*k* = −14→14
*T*_min_ = 0.942, *T*_max_ = 1.000	*l* = −15→15
12190 measured reflections	

### Refinement

**Table:**

Refinement on *F*^2^	Primary atom site location: dual
Least-squares matrix: full	Hydrogen site location: mixed
*R*[*F*^2^ > 2σ(*F*^2^)] = 0.020	H atoms treated by a mixture of independent and constrained refinement
*wR*(*F*^2^) = 0.052	*w* = 1/[σ^2^(*F*_o_^2^) + (0.0288*P*)^2^ + 0.2552*P*] where *P* = (*F*_o_^2^ + 2*F*_c_^2^)/3
*S* = 1.07	(Δ/σ)_max_ = 0.004
4404 reflections	Δρ_max_ = 0.78 e Å^−^^3^
208 parameters	Δρ_min_ = −0.29 e Å^−^^3^
0 restraints	

### Special details

**Table:**

Geometry. All esds (except the esd in the dihedral angle between two l.s. planes) are estimated using the full covariance matrix. The cell esds are taken into account individually in the estimation of esds in distances, angles and torsion angles; correlations between esds in cell parameters are only used when they are defined by crystal symmetry. An approximate (isotropic) treatment of cell esds is used for estimating esds involving l.s. planes.
Refinement. The positions of the N-bound and the O-bound H atoms were obtained from difference Fourier maps and were refined isotropically. The C-bound H atoms were placed at ideal positions and were refined as riding on their parent C atoms. *U*_iso_(H) values of the H atoms were set at 1.2*U*_eq_(parent atom for C_sp2_) and 1.5*U*_eq_(parent atom for C_sp3_).

### Fractional atomic coordinates and isotropic or equivalent isotropic displacement parameters (Å^2^)

**Table:**

	*x*	*y*	*z*	*U*_iso_*/*U*_eq_	
I1	0.41685 (2)	0.80629 (2)	0.76854 (2)	0.03537 (5)	
N1	0.38277 (16)	0.22734 (11)	0.80569 (10)	0.0189 (2)	
N2	0.22902 (17)	0.60900 (11)	0.53955 (11)	0.0206 (2)	
N3	0.24819 (18)	0.43530 (12)	0.39835 (11)	0.0220 (2)	
C6	0.19028 (19)	0.69330 (13)	0.45019 (12)	0.0202 (2)	
C4	0.3287 (2)	0.25792 (13)	0.60271 (13)	0.0221 (3)	
H3	0.3186	0.2239	0.5269	0.026*	
C3	0.30663 (18)	0.38741 (12)	0.62082 (12)	0.0181 (2)	
C2	0.3276 (2)	0.43318 (13)	0.73619 (13)	0.0217 (3)	
H2	0.3151	0.5189	0.7522	0.026*	
C1	0.3666 (2)	0.35186 (13)	0.82613 (13)	0.0219 (3)	
H1	0.3820	0.3835	0.9019	0.026*	
C5	0.3656 (2)	0.17955 (13)	0.69587 (13)	0.0222 (3)	
H4	0.3785	0.0934	0.6826	0.027*	
C16	0.4153 (2)	0.14270 (14)	0.90731 (13)	0.0233 (3)	
H13	0.4977	0.1841	0.9559	0.028*	
H14	0.4801	0.0667	0.8733	0.028*	
C15	0.18123 (18)	0.64498 (13)	0.33129 (12)	0.0197 (2)	
C14	0.2089 (2)	0.51456 (14)	0.30483 (13)	0.0218 (3)	
C7	0.1655 (2)	0.82045 (14)	0.47347 (14)	0.0267 (3)	
H6	0.1693	0.8515	0.5514	0.032*	
C10	0.14939 (19)	0.73002 (14)	0.23651 (13)	0.0232 (3)	
C11	0.1474 (2)	0.68118 (17)	0.11772 (14)	0.0292 (3)	
H9	0.1297	0.7353	0.0543	0.035*	
C9	0.1257 (2)	0.85948 (15)	0.26407 (14)	0.0275 (3)	
H8	0.1040	0.9157	0.2033	0.033*	
C8	0.1343 (2)	0.90312 (15)	0.37892 (16)	0.0299 (3)	
H7	0.1193	0.9888	0.3949	0.036*	
C13	0.2006 (2)	0.47006 (16)	0.18876 (14)	0.0296 (3)	
H11	0.2143	0.3845	0.1721	0.035*	
C12	0.1712 (2)	0.55533 (18)	0.09582 (14)	0.0327 (3)	
H10	0.1680	0.5250	0.0175	0.039*	
C17	0.2286 (2)	0.10846 (19)	0.98868 (17)	0.0362 (4)	
H17	0.1531	0.0588	0.9431	0.054*	
H15	0.1592	0.1838	1.0168	0.054*	
H16	0.2543	0.0610	1.0580	0.054*	
B1	0.2602 (2)	0.47939 (14)	0.51714 (14)	0.0189 (3)	
H5	0.246 (3)	0.639 (2)	0.604 (2)	0.035 (6)*	
H12	0.280 (3)	0.365 (2)	0.373 (2)	0.035 (5)*	

### Atomic displacement parameters (Å^2^)

**Table:**

	*U*^11^	*U*^22^	*U*^33^	*U*^12^	*U*^13^	*U*^23^
I1	0.06050 (9)	0.01998 (6)	0.02905 (6)	0.00500 (4)	−0.01733 (5)	−0.00322 (4)
N1	0.0171 (5)	0.0176 (5)	0.0217 (5)	0.0001 (4)	−0.0027 (4)	0.0065 (4)
N2	0.0239 (6)	0.0190 (5)	0.0187 (5)	0.0031 (4)	−0.0037 (4)	0.0041 (4)
N3	0.0252 (6)	0.0176 (5)	0.0238 (6)	0.0014 (4)	−0.0063 (4)	0.0032 (4)
C6	0.0177 (6)	0.0196 (6)	0.0224 (6)	0.0025 (5)	−0.0016 (5)	0.0066 (5)
C4	0.0259 (7)	0.0191 (6)	0.0216 (6)	−0.0010 (5)	−0.0053 (5)	0.0034 (5)
C3	0.0148 (6)	0.0175 (6)	0.0210 (6)	−0.0001 (4)	−0.0007 (4)	0.0056 (4)
C2	0.0251 (7)	0.0160 (6)	0.0232 (6)	0.0015 (5)	−0.0028 (5)	0.0037 (5)
C1	0.0261 (7)	0.0186 (6)	0.0210 (6)	0.0018 (5)	−0.0041 (5)	0.0019 (5)
C5	0.0256 (7)	0.0148 (6)	0.0264 (6)	0.0002 (5)	−0.0049 (5)	0.0035 (5)
C16	0.0238 (7)	0.0212 (6)	0.0259 (7)	0.0008 (5)	−0.0076 (5)	0.0099 (5)
C15	0.0146 (6)	0.0225 (6)	0.0210 (6)	0.0010 (5)	−0.0008 (4)	0.0067 (5)
C14	0.0187 (6)	0.0241 (7)	0.0225 (6)	0.0007 (5)	−0.0041 (5)	0.0047 (5)
C7	0.0313 (8)	0.0207 (6)	0.0281 (7)	0.0047 (5)	−0.0056 (6)	0.0039 (5)
C10	0.0152 (6)	0.0293 (7)	0.0242 (6)	0.0017 (5)	−0.0012 (5)	0.0107 (5)
C11	0.0222 (7)	0.0418 (9)	0.0233 (7)	0.0048 (6)	−0.0037 (5)	0.0107 (6)
C9	0.0224 (7)	0.0278 (7)	0.0309 (7)	0.0028 (6)	−0.0012 (5)	0.0154 (6)
C8	0.0304 (8)	0.0211 (7)	0.0372 (8)	0.0047 (6)	−0.0041 (6)	0.0097 (6)
C13	0.0326 (8)	0.0308 (8)	0.0270 (7)	0.0049 (6)	−0.0100 (6)	−0.0013 (6)
C12	0.0317 (8)	0.0459 (10)	0.0218 (7)	0.0072 (7)	−0.0084 (6)	0.0005 (6)
C17	0.0270 (8)	0.0448 (10)	0.0364 (8)	−0.0022 (7)	−0.0053 (6)	0.0246 (7)
B1	0.0158 (6)	0.0184 (6)	0.0221 (7)	−0.0008 (5)	−0.0026 (5)	0.0060 (5)

### Geometric parameters (Å, º)

**Table:**

N1—C1	1.3446 (18)	C16—H14	0.9700
N1—C5	1.3452 (19)	C16—C17	1.506 (2)
N1—C16	1.4855 (16)	C15—C14	1.423 (2)
N2—C6	1.3930 (16)	C15—C10	1.4283 (18)
N2—B1	1.4100 (19)	C14—C13	1.382 (2)
N2—H5	0.81 (2)	C7—H6	0.9300
N3—C14	1.3961 (17)	C7—C8	1.409 (2)
N3—B1	1.415 (2)	C10—C11	1.418 (2)
N3—H12	0.81 (2)	C10—C9	1.413 (2)
C6—C15	1.427 (2)	C11—H9	0.9300
C6—C7	1.381 (2)	C11—C12	1.365 (3)
C4—H3	0.9300	C9—H8	0.9300
C4—C3	1.3957 (19)	C9—C8	1.367 (2)
C4—C5	1.3816 (18)	C8—H7	0.9300
C3—C2	1.4012 (19)	C13—H11	0.9300
C3—B1	1.5808 (19)	C13—C12	1.410 (2)
C2—H2	0.9300	C12—H10	0.9300
C2—C1	1.3786 (18)	C17—H17	0.9600
C1—H1	0.9300	C17—H15	0.9600
C5—H4	0.9300	C17—H16	0.9600
C16—H13	0.9700		
			
C1—N1—C5	120.68 (11)	C14—C15—C10	119.66 (13)
C1—N1—C16	118.97 (12)	N3—C14—C15	117.96 (13)
C5—N1—C16	120.33 (12)	C13—C14—N3	121.88 (14)
C6—N2—B1	122.92 (12)	C13—C14—C15	120.15 (13)
C6—N2—H5	116.8 (16)	C6—C7—H6	120.0
B1—N2—H5	119.9 (16)	C6—C7—C8	119.91 (15)
C14—N3—B1	122.71 (13)	C8—C7—H6	120.0
C14—N3—H12	112.2 (16)	C11—C10—C15	118.47 (14)
B1—N3—H12	124.4 (16)	C9—C10—C15	118.79 (14)
N2—C6—C15	117.93 (12)	C9—C10—C11	122.73 (13)
C7—C6—N2	121.80 (13)	C10—C11—H9	119.8
C7—C6—C15	120.25 (12)	C12—C11—C10	120.44 (14)
C3—C4—H3	119.5	C12—C11—H9	119.8
C5—C4—H3	119.5	C10—C9—H8	119.6
C5—C4—C3	120.92 (13)	C8—C9—C10	120.85 (13)
C4—C3—C2	116.81 (12)	C8—C9—H8	119.6
C4—C3—B1	122.23 (12)	C7—C8—H7	119.5
C2—C3—B1	120.96 (12)	C9—C8—C7	121.07 (15)
C3—C2—H2	119.8	C9—C8—H7	119.5
C1—C2—C3	120.39 (13)	C14—C13—H11	120.2
C1—C2—H2	119.8	C14—C13—C12	119.51 (15)
N1—C1—C2	120.87 (13)	C12—C13—H11	120.2
N1—C1—H1	119.6	C11—C12—C13	121.73 (15)
C2—C1—H1	119.6	C11—C12—H10	119.1
N1—C5—C4	120.31 (13)	C13—C12—H10	119.1
N1—C5—H4	119.8	C16—C17—H17	109.5
C4—C5—H4	119.8	C16—C17—H15	109.5
N1—C16—H13	109.4	C16—C17—H16	109.5
N1—C16—H14	109.4	H17—C17—H15	109.5
N1—C16—C17	111.19 (12)	H17—C17—H16	109.5
H13—C16—H14	108.0	H15—C17—H16	109.5
C17—C16—H13	109.4	N2—B1—N3	117.28 (12)
C17—C16—H14	109.4	N2—B1—C3	121.20 (12)
C6—C15—C10	119.13 (13)	N3—B1—C3	121.51 (12)
C14—C15—C6	121.19 (12)		
			
N2—C6—C15—C14	0.62 (19)	C16—N1—C1—C2	−176.98 (12)
N2—C6—C15—C10	−177.51 (12)	C16—N1—C5—C4	177.77 (13)
N2—C6—C7—C8	177.40 (14)	C15—C6—C7—C8	−1.1 (2)
N3—C14—C13—C12	−176.41 (14)	C15—C14—C13—C12	2.2 (2)
C6—N2—B1—N3	−0.9 (2)	C15—C10—C11—C12	1.4 (2)
C6—N2—B1—C3	179.14 (12)	C15—C10—C9—C8	0.5 (2)
C6—C15—C14—N3	−0.94 (19)	C14—N3—B1—N2	0.5 (2)
C6—C15—C14—C13	−179.61 (13)	C14—N3—B1—C3	−179.49 (12)
C6—C15—C10—C11	177.85 (12)	C14—C15—C10—C11	−0.31 (19)
C6—C15—C10—C9	−0.70 (19)	C14—C15—C10—C9	−178.86 (13)
C6—C7—C8—C9	0.8 (2)	C14—C13—C12—C11	−1.1 (3)
C4—C3—C2—C1	−0.5 (2)	C7—C6—C15—C14	179.15 (13)
C4—C3—B1—N2	176.79 (13)	C7—C6—C15—C10	1.0 (2)
C4—C3—B1—N3	−3.2 (2)	C10—C15—C14—N3	177.18 (12)
C3—C4—C5—N1	−0.7 (2)	C10—C15—C14—C13	−1.5 (2)
C3—C2—C1—N1	−0.9 (2)	C10—C11—C12—C13	−0.7 (2)
C2—C3—B1—N2	−3.24 (19)	C10—C9—C8—C7	−0.5 (2)
C2—C3—B1—N3	176.80 (13)	C11—C10—C9—C8	−178.03 (15)
C1—N1—C5—C4	−0.7 (2)	C9—C10—C11—C12	179.89 (15)
C1—N1—C16—C17	84.82 (17)	B1—N2—C6—C15	0.33 (19)
C5—N1—C1—C2	1.6 (2)	B1—N2—C6—C7	−178.18 (13)
C5—N1—C16—C17	−93.72 (17)	B1—N3—C14—C15	0.3 (2)
C5—C4—C3—C2	1.3 (2)	B1—N3—C14—C13	178.99 (14)
C5—C4—C3—B1	−178.72 (13)	B1—C3—C2—C1	179.52 (13)

### Hydrogen-bond geometry (Å, º)

**Table:**

*D*—H···*A*	*D*—H	H···*A*	*D*···*A*	*D*—H···*A*
N2—H5···I1	0.81 (2)	2.96 (2)	3.7260 (13)	157.2 (18)
C2—H2···I1	0.93	3.16 (1)	4.0483 (14)	161 (1)
N3—H12···I1^i^	0.82 (2)	3.02 (2)	3.7453 (12)	148.7 (18)
C16—H14···I1^ii^	0.97	3.07 (1)	3.8981 (15)	144 (1)

Symmetry codes: (i) −*x*+1, −*y*+1, −*z*+1; (ii) *x*, *y*−1, *z*.
